# TIME HELP FROM ADULT CHILDREN: DIFFERENCES AMONG BIOLOGICAL AND STEP-CHILDREN IN NON-STEPFAMILIES AND STEPFAMILIES

**DOI:** 10.1093/geroni/igae098.1767

**Published:** 2024-12-31

**Authors:** I-Fen Lin, Judith Seltzer, Janecca Chin, Emily Wiemers, Anna Wiersma Strauss, V Hotz

**Affiliations:** Bowling Green State University, Bowling Green, Ohio, United States; University of California Los Angeles, Los Angeles, California, United States; Bowling Green State University, Bowling Green, Ohio, United States; Syracuse University, Syracuse, New York, United States; Syracuse University, Syracuse, New York, United States; Duke University, Durham, North Carolina, United States

## Abstract

Objectives. Adult stepchildren help their parents less than biological children, but findings based on parent-child dyads ignore that some biological children are in stepfamilies because one or both parents have children from previous unions. Additionally, little is known about how a widespread crisis may disrupt the normal patterns of assistance from adult children to parents. We take a family systems approach to investigate whether the COVID-19 pandemic reshaped adult children’s time help to parents by considering dyadic relationships embedded in family structure. Methods. We leveraged the panel design of the Health and Retirement Study to examine changes in time help to parents. We compared adult children’s responses to parental need prior to and during the pandemic using the 2016, 2018, and 2020 core interviews. We also examined how children responded to parental need using the 2020 COVID-19 module. Throughout we considered biological children and stepchildren in non-stepfamilies and stepfamilies. Results. Stepchildren were least likely to provide time help, followed by biological children in stepfamilies, and lastly biological children in non-stepfamilies. However, among parents who had trouble with instrumental activities of daily living (2016, 2018, 2020 core interviews) or had trouble buying food for non-financial reasons (COVID-19 module), biological children in non-stepfamilies and biological children in stepfamilies were equally likely to help their parents. Discussion. Our findings suggest general stability in biological versus step children’ helping differentials even in times of crisis but also challenge the view that the presence of a stepchild weakens ties between biological children and their parents.

